# Use of an Integrated Pan-Cancer Oncology Enrichment Next-Generation Sequencing Assay to Measure Tumour Mutational Burden and Detect Clinically Actionable Variants

**DOI:** 10.1007/s40291-020-00462-x

**Published:** 2020-04-18

**Authors:** Valerie Pestinger, Matthew Smith, Toju Sillo, John M. Findlay, Jean-Francois Laes, Gerald Martin, Gary Middleton, Phillipe Taniere, Andrew D. Beggs

**Affiliations:** 1 Surgical Research Laboratory, Institute of Cancer and Genomic Sciences, University of Birmingham, Vincent Drive, Birmingham, B15 2TT UK; 2 Queen Elizabeth Hospital Birmingham, Birmingham, UK; 3 Northern Devon Healthcare NHS Trust, Barnstaple, UK; 4 OncoDNA, Gosselies, Belgium; 5 PierianDx, St. Louis, MO USA; 6 Institute of Immunology and Immunotherapy, University of Birmingham, Birmingham, UK

## Abstract

**Introduction:**

The identification of tumour mutational burden (TMB) as a biomarker of response to programmed cell death protein 1 (PD-1) immunotherapy has necessitated the development of genomic assays to measure this. We carried out comprehensive molecular profiling of cancers using the Illumina TruSight Oncology 500 (TSO500) panel and compared these to whole-genome sequencing (WGS).

**Methods:**

Cancer samples derived from formalin-fixed material were profiled on the TSO500 panel, sequenced on an Illumina NextSeq 500 instrument and processed through the TSO500 Docker pipeline. Either FASTQ files (PierianDx) or vcf files (OncoKDM) were processed to understand clinical actionability.

**Results:**

In total, 108 samples (a mixture of colorectal, lung, oesophageal and control samples) were processed via the DNA panel. There was good correlation between TMB, single-nucleotide variants (SNVs), indels and copy-number variations as predicted by TSO500 and WGS (*R*^2^ > 0.9) and good reproducibility, with less than 5% variability between repeated controls. For the RNA panel, 13 samples were processed, with all known fusions observed via orthogonal techniques. For clinical actionability, 72 tier 1 variants and 297 tier 2 variants were detected, with clinical trials identified for all patients.

**Conclusions:**

The TSO500 assay accurately measures TMB, microsatellite instability, SNVs, indels, copy-number/structural variation and gene fusions when compared to WGS and orthogonal technologies. Coupled with a clinical annotation pipeline, this provides a powerful methodology for identification of clinically actionable variants.

**Electronic supplementary material:**

The online version of this article (10.1007/s40291-020-00462-x) contains supplementary material, which is available to authorized users.

## Key Points

**Table:** 

Formalin-fixed, paraffin-embedded tumour samples represent a significant diagnostic challenge for diagnostic analysis in molecular pathology
The Illumina TruSight Oncology 500 assay provides an option to detect relevant cancer gene mutations and fusions
These variants can also be annotated easily with clinical interpretation software for ease of clinical interpretation

## Introduction

Recent developments in next-generation sequencing (NGS) and tumour immunology have allowed the discovery that targeting the CTLA4, programmed cell death protein 1 (PD-1) and programmed death-ligand 1 (PD-L1) receptors using therapeutic monoclonal antibodies [1] can unmask cancer to the immune system, facilitating its immune-mediated destruction. Although initial trials of PD-1 inhibitors had mixed results [2, 3], as with previous targeted therapies, it was determined that a specific tumour genotype was required in order for these inhibitors to be effective, leading to the finding that dramatic regression of tumours could occur with the correct genotype.

In order for tumours to become immunogenic, a high neoepitope load must be generated via hypermutation [4–6], ideally indel/frameshifts or non-synonymous mutations that generate novel proteins that can be recognised by the immune system. These neoepitopes can then be presented via major histocompatibility complex (MHC) in order to aid immune killing [7].

The CHECKMATE [8–10] series of trials have suggested that a specific threshold of “tumour mutational burden” (TMB) must be reached in order for PD-1 blockade to become effective, although this has not been adopted formally, due to a lack of association between TMB and response, although other similar markers such as clonal TMB have shown promise. Although TMB has variable definitions, it is broadly accepted [9] as the number of missense mutations in the tumour genome, either divided by the size of the exome panel (35–45 Mb) or via the size of the human genome for whole-genome sequencing (WGS) (3.3 Gb). Based on the CHECKMATE trials, the suggested TMB threshold is greater than 10 mutations (mut)/Mb, based on the objective response rates of the tumours in these studies not improving much beyond this threshold.

Initially, TMB was measured using WGS and whole-exome sequencing [11]; however, these technologies are not cost-effective currently for routine use in the clinic. Despite the falling cost of NGS reagents, the volume of data required for sufficient coverage of either WGS (200–300 Gb for 60× read depth) or whole-exome sequencing (4–5 Gb for 100× read depth) make these techniques impractical except for dedicated sequencing cores. Secondly, even with high read depth, sufficiently deep coverage in order to identify rare subclonal [12] mutations that may contribute to the neoantigen load is required, of the order of 500×. Thus, whole-genome/exome coverage is not cost-effective [13].

Rizvi et al. [13] demonstrated that in order to accurately measure TMB using an NGS-based assay, a panel size of at least 1.5 Mb is required. This panel size offers opportunities for a pan-cancer assay, as a panel of this size could cover the majority of known driver genes across multiple cancer types. In designing an oncology assay, ideally other types of variations would be included. Recent studies [14, 15] have shown the potential utility of selecting targeted therapies using large gene panels, and therefore a panel should include mutations associated with targeting therapies.

An additional advantage of panel-based designs is the ability to enrich RNA targets. Recent studies have shown the importance of RNA fusions such as the *TMPRSS-ERG* fusion in prostate cancer [16], the *FGFR2* fusion in cholangiocarcinoma [17] and the *NTRK* fusion in lung and other cancers [18]. These fusions are either targetable with molecularly targeted agents (e.g. larotrectinib [19] or pemigatinib [20]) or are prognostically relevant (i.e. *TMPRSS-ERG*).

An ideal oncology panel-based assay would have several characteristics [21]: enrichment chemistry rather than polymerase chain reaction (PCR) chemistry for identification of rare alleles with straightforward library preparation; a broad panel that targets the majority of DNA and RNA alterations in cancer; rapid run time; prediction of novel biomarkers such as TMB; and a standardised, reproducible analysis pipeline that can be used in a clinical setting.

In this study, we present our initial results using the Illumina TruSight Oncology 500 (TSO500) assay across a range of cancer types. We benchmarked it against WGS and whole-exome sequencing and determined its ability to detect RNA fusions and copy-number variants.

## Materials and Methods

### Patient Samples and Ethics

Patient samples were from three cohorts—colorectal, oesophageal and lung—in order to provide a variety of mutations for study across multiple tissue types. For the colorectal cohort, fresh frozen tumours were obtained from an internal cohort of patients that had undergone WGS as part of a pre-pilot prior to the introduction of the 100,000 Genomes project. Matched formalin-fixed, paraffin-embedded (FFPE) blocks from the tumour were obtained and used for the assay. For the oesophageal cohort, sequential oesophageal cancers underwent whole-exome sequencing from a cohort collected at the Oxford Cancer Centre. For the lung cohort, patient samples were obtained from those undergoing routine testing for *EGFR* mutation status at the Molecular Pathology department of the University Hospitals Birmingham NHS Foundation Trust.

Ethics approval for the study was obtained from the Oxford Ethics committee (reference 05/Q1605/66).

### Nucleic Acid Extractions and Quality Assessment

DNA and RNA were extracted from 2 × 5-µm FFPE scrolls on the Covaris E220 evolution (520220, Covaris Ltd, Woodingdean, Brighton, UK) using the truXTRAC FFPE total NA Kit—Column Purification (520220, Covaris Ltd, Woodingdean, Brighton, UK) following the manufacturer’s protocol.

Sixty-five per cent isopropanol was used during RNA purification. On-column DNA digestion was performed after the first wash during RNA purification using the TURBO DNA-free kit (AM1907, Invitrogen, ThermoFisher Scientific, Paisley, UK) following the Covaris protocol. DNA and RNA concentrations were measured on the Qubit 3 Fluorometer (ThermoScientific, Paisley, UK) and the percentage of fragments > 200 nucleotides in size (DV200) was assessed using Tapestation 2200 (Agilent, Cheshire, UK). DNA quality was determined by the Infinium HD FFPE quality control (QC) Assay Protocol (15020981, Illumina, Cambridge, UK).

RNA samples with a DV200 of ≥ 30% and DNA samples with a Delta Cq value of ≤ 5 were used for downstream applications.

### Library Preparation

DNA libraries were prepared using the hybrid capture-based TruSight Oncology 500 Library Preparation Kit (Illumina, San Diego, CA, USA) following Illumina’s TruSight Oncology 500 Reference Guide (document # 1000000067621 v00, Illumina Cambridge, UK) with the following modifications:

Genomic DNA (gDNA) was sheared using the Covaris E220 evolution (Covaris Ltd, Woodingdean, Brighton, UK), 8 micro TUBE—50 AFA Fiber Strip V2 (520174, Covaris Ltd, Woodingdean, Brighton, UK) and Rack E220e 8 microTUBE Strip V2 (500437, Covaris Ltd, Woodingdean, Brighton, UK). The size of double-stranded DNA (dsDNA) fragments (90–250 bp) was confirmed using Tapestation 2200 (Agilent, Cheshire, UK) after shearing. A HorizonDx HD753 control (Horizon Discovery, Cambridge, UK) was included with every set of seven test samples. When no beads were involved, reagents were mixed by pipetting up and down ten times. Before the bead-based normalisation, libraries were quantified and sized on the Qubit 3 Fluorometer (ThermoScientific, Paisley, UK) and Tapestation 2200 (Agilent, Cheshire, UK), respectively.

Ten microlitres of each normalised DNA library (maximum of eight libraries per pool) was pooled and incubated at 96 °C for 2 min. The tube containing the library pool was immediately inverted two times to mix, centrifuged briefly and placed on ice for 5 min. Ten microlitres of the library pool was mixed with 190 µL HT1 to make a 1:20 dilution (DIL1). Forty microlitres of DIL1 were mixed with 1360 µL HT1 (for a final library concentration of 1.5 pM), and 2.5 µL of denatured 20 pM PhiX was added (1%). Libraries were sequenced on an Illumina NextSeq 500 instrument.

For WGS libraries, 1 µg of DNA was prepared using the TruSeq DNA library preparation kit (Illumina, San Diego, CA, USA) and sequenced across four lanes of a HiSeq 2500 (Illumina, San Diego, CA, USA).

### Bioinformatics

The raw sequencing output was transferred from the sequencing instrument to a bioinformatics server running Ubuntu 18.04LTS. A pre-supplied Docker image (the TSO500 pipeline; Illumina, San Diego, CA, USA) was used to generate TMB and microsatellite instability (MSI) calls. The pipeline consists of several steps. Initially, raw bcl files were converted to sample-specific FASTQ files as specified by the sample index. FASTQ files were then aligned against the hg19 reference genome using Isaac 4; local realignment to indels was performed, and paired-end reads were stitched together, followed by variant calling with the somatic sample caller Pisces. Germline variants were filtered using a proprietary database; then the called variants were annotated to identify synonymous and non-synonymous variants. Actual coverage of the panel compared to the reference coverage was computed, and TMB was calculated based on the number of synonymous and non-synonymous mutations detected divided by the size of the panel successfully sequenced.

Small variants were exported from the TSO500 pipeline and annotated using VEP, then converted using vcf2maf and imported into the maftools module of R/Bioconductor.

TMB calls for whole-genome sequenced control data were carried out using the Genomics England v3 pipeline for calling tumour-normal pairs and used to compare to calls from the TSO500 pipeline. In brief, this pipeline utilised Isaac v3 to align sequence data to the hg19 genome, followed by copy-number variant calling using Canvas and structural variant calling using Manta. Copy-number variation (CNV) calls for the TSO500 files were obtained using the Craft copy-number caller set in somatic tumour only mode. Overlaps were computed using bedtools. Structural variant calls for the TSO500 files were obtained using the Manta structural variant caller set in tumour only mode with a custom modification to the C++ code of the Manta structural variant caller to enable detection with less read support and on amplicon sequencing data. Structural variant overlaps were computed using bedtools.

For clinical actionability, raw FASTQ files (CGW, PierianDx, St. Louis, MO, USA) and UMI collapsed vcf files obtained from the TSO500 v1 Docker image (OncoKDM, OncoDNA, Gosselies, Belgium) were uploaded to their respective data portals and run in their standard analysis mode. The Clinical Genomic Workspace (CGW; PierianDx, St Louis, MO, USA) is a secure web-based Health Insurance Portability and Accountability Act- and General Data Protection Regulations-compliant platform for clinical decision support management. Initially developed by one of the very first medical institutes to launch a routine clinical NGS service for cancer and complex inherited diseases, the CGW encompasses a rules engine built on a curated knowledgebase that is updated weekly. Information from over 18 million publications, including Food and Drug Administration (FDA) and European Medicines Agency (EMA) approvals, National Comprehensive Cancer Network (NCCN), Association of Molecular Pathology (AMP) and European Society for Molecular Oncology (ESMO) guidelines and PubMed articles is coupled with public data sources such as population databases, dbSNP, The Cancer Genome Atlas (TCGA), ClinVar and COSMIC in order to annotate and pre-classify variants for interpretation. Uniquely, the CGW utilises the world's largest clinical interpretation-sharing network that provides variant interpretations in the context of the specific disease defined for the patient at time of accessioning. Although no patient data are transferred, network members can view the clinical interpretations supplied to the clinical team of the provider institution (giving the most up-to-date information with true clinical provenance). Actionability calls were downloaded according to standard AMP tiers. The CGW platform is configurable to accept bcl, FASTQ or vcf files and can process all variant types, including TMB and MSI biomarkers, complex variants, CNVs and fusions.

OncoKDM is a secure web-based ISO27001, IS013485 and GDPR-compliant platform for clinical decision support management and clinical report sharing. Initially developed for its proprietary OncoDEEP products that have been on the market since 2013, OncoKDM encompasses a proprietary daily/weekly curated knowledge database of 22,000 genes, 3,886,000 variants, 792 drugs (including FDA and EMA approvals, NCCN, Compermed and ESMO guidelines), 5000 associated clinical trials and 7000 associated publications. Coupled with several public data sources, OncoKDM accurately retrieves biological and clinical information for proper data interpretation and has already been used for 6 years thanks to the sharing platform OncoSHARE, used by 6500 healthcare professionals in 50 countries worldwide.

## Results

### DNA Quality Metrics

In total, 108 samples were profiled using the assay, with a median sample age of 2 years (range 4 months–10 years). All samples were from FFPE blocks. All samples were examined by haematoxylin staining and had tumour content > 50%. The input for all assays was 40 ng of DNA and 40 ng of RNA. The suitability of samples for sequencing was determined using the real-time PCR-based Illumina FFPE QC assay. For a sample to pass initial QC, the delta Cycle threshold (dCt) must be under 5. Study samples had a median dCt of 2.46 [interquartile range (IQR) 1.73–4.3]. The maximum dCt run was 13.57, and all samples bar one passed TMB calling. Eight samples failed MSI calling due to poor sample quality (dCt = 5–13.57). Samples were all sequenced despite the initial QC metrics to determine the validity of this measure in determining samples for sequencing.

In terms of DNA sequencing metrics, the median insert size was 92.5 bp (IQR 80–112 bp), the median exon coverage was 185× (IQR 123–247×), and 98.1% and 90.5% of all samples were covered at least 50× and 100×, respectively. The median reads per sample was 126 M (IQR 105–138 M reads); there was a median of 0.9% (IQR 0.25–2.7%) chimeric reads per sample. Median read enrichment was 82.1% (IQR 79–85%).

### Mutational Coverage and Spectrum

**Fig. 1:**
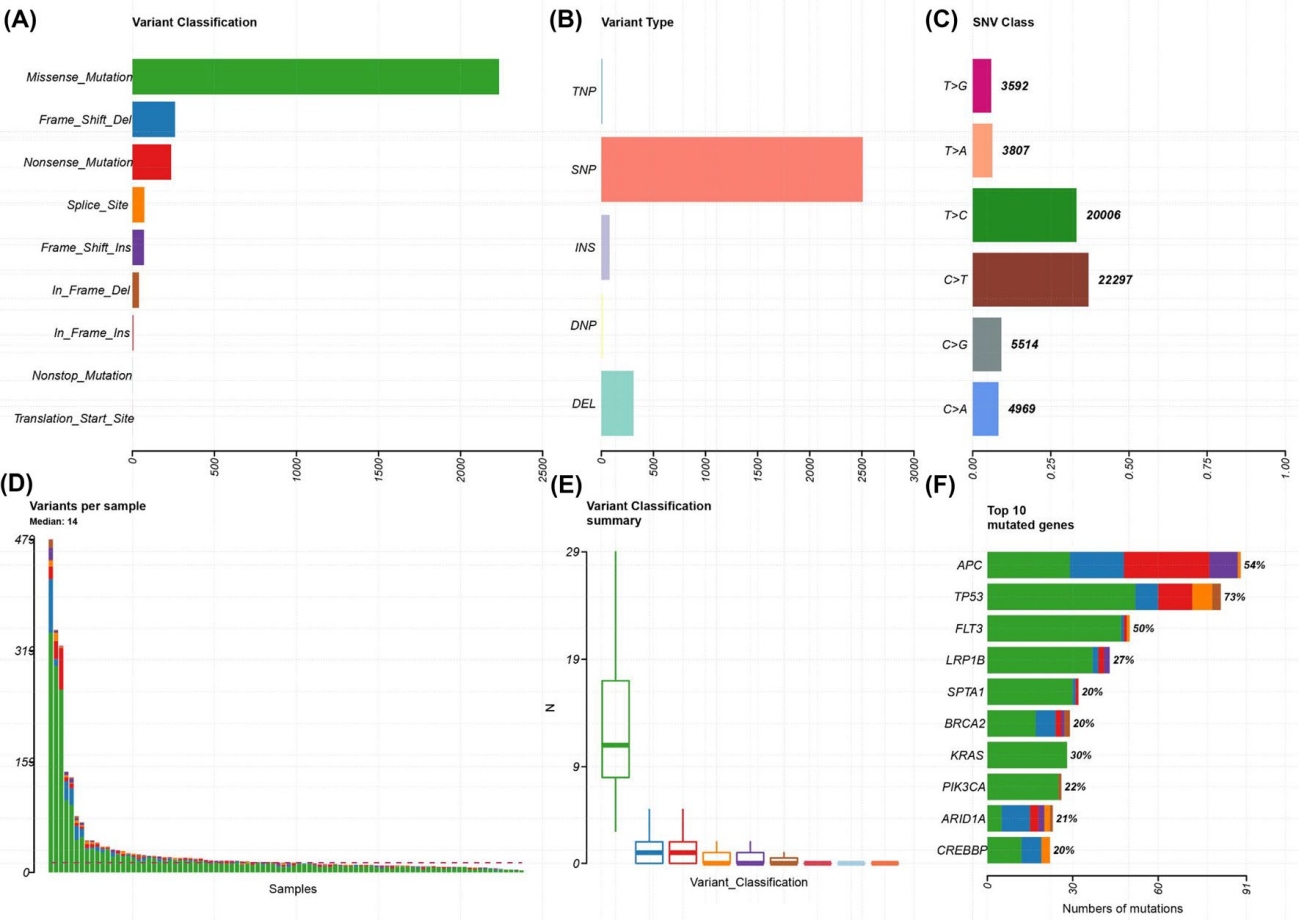
Plot of variant classification for all samples using TSO500. **a** Total number of variants detected by variant classification. **b** Total number of variants detected by variant type. **c** Total number of variants detected by SNV class. **d** Number of variants per sample. **e** Box plot of the number of variants within each classification per sample. **f** Top 10 mutated genes. For **d**–**f**, the colours are equivalent to **a**. *DEL* deletion, *DNP* dinucleotide polymorphism, *INS* insertion, *SNP* single-nucleotide polymorphism, *SNV* single-nucleotide variant, *TNP* trinucleotide polymorphism, *TSO500* TruSight Oncology 500

**Fig. 2:**
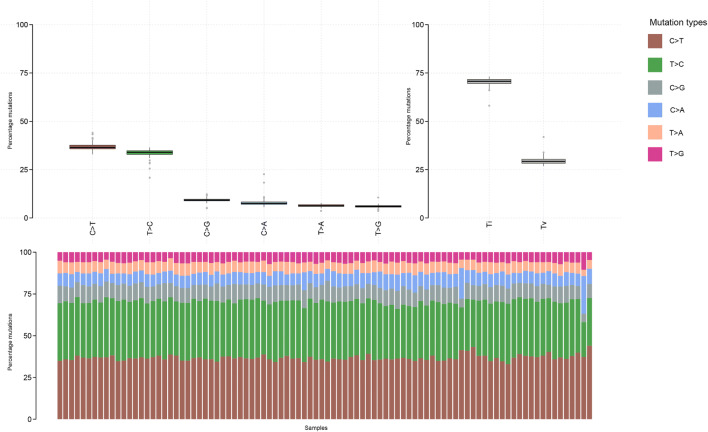
Mutational plot for all samples in TSO500 assay. Top left panel: Type of mutation (frequency in percent). Top right panel: Transitions (Ti) vs. transversion (Tv). Bottom panel: Proportion of mutations. *TSO500* TruSight Oncology 500

**Fig. 3:**
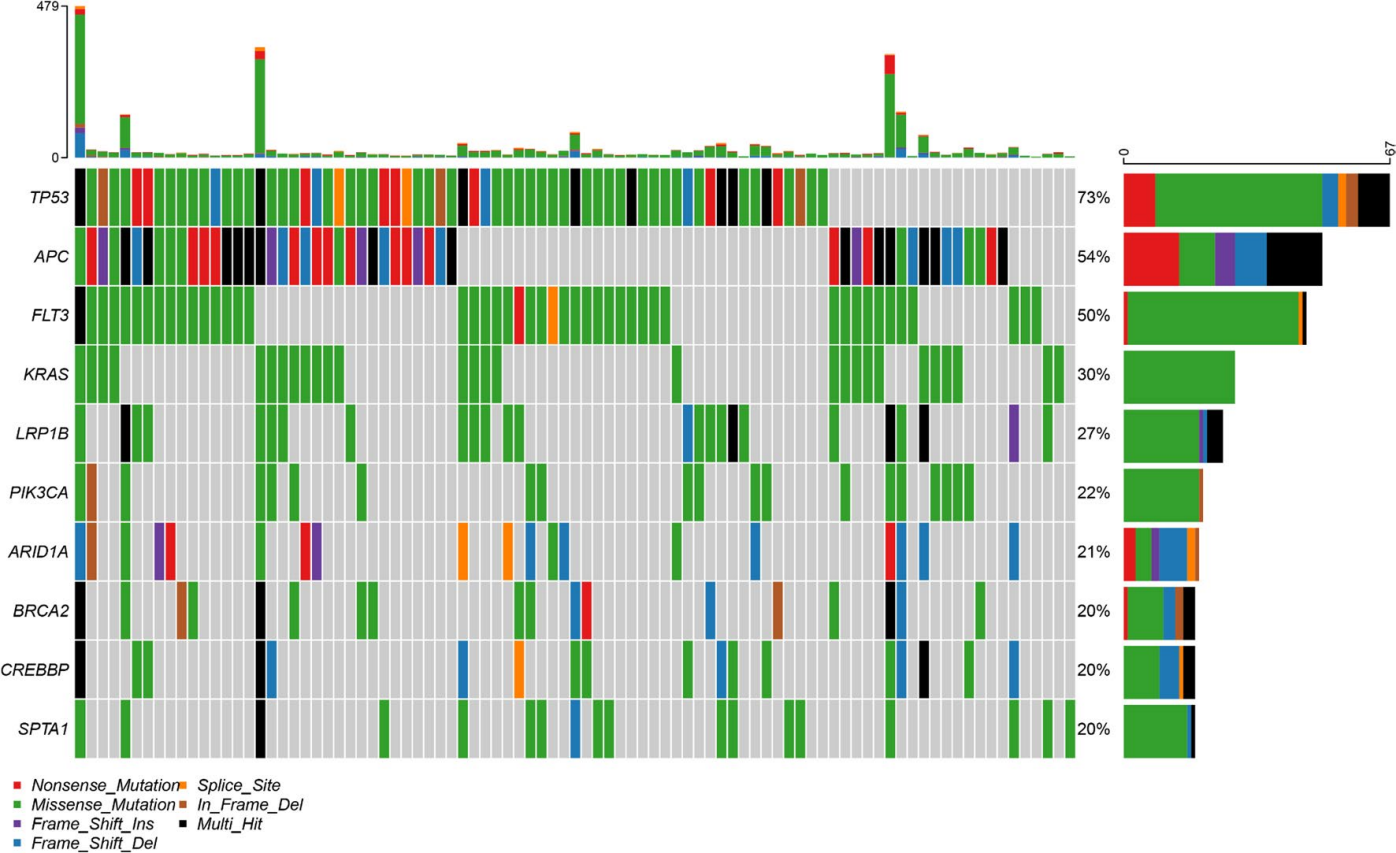
OncoStrip plot of common variants across samples. Genes on *y*-axis. Samples on *x*-axis

In all 108 samples, mutational coverage of the panel was successfully performed, with little probe drop out. Figures 1, 2 and 3 show the variant classification results (for cancer type variant results, see supplementary Fig. 1 in the electronic supplementary material). There was a median of 14 variants per sample (range 2–479). The predominant mutation type was a missense single-nucleotide polymorphism (SNP), followed by frameshift deletion. The top ten most commonly mutated genes were *TP53* (73%), *APC* (54%), *FLT3* (50%), *LRP1B* (27%), *SPTA1* (20%), *BRCA2* (20%), *KRAS* (30%), *PIK3CA* (22%), *ARID1A* (21%) and *CREBBP* (20%). Within the sample subgroups, colorectal cancer was significantly enriched for *APC* mutations (40/54 colorectal cancer samples), *ZFHX3* (13/54 samples) and *FBXW7* (13/54). Oesophageal cancer was significantly enriched for *EPHA7* mutations (3/9 samples) and TP53 (9/9 samples). For lung, *RBM10* (5/22 samples) and *MGA* (7/22 samples) were significantly enriched.

In terms of mutational spectrum, a predominance of C > T transversions was seen, as the samples were all derived (with the exception of controls) from FFPE tissue blocks. The predominant mutational signatures [22] seen in the samples were signature ten (defects in polymerase *POLE*), signature five (due to tobacco smoke) and signature six (defective mismatch repair), which fit well with the source of the samples (lung, colorectal and oesophageal) as well as the fact that several hypermutant samples were deliberately chosen for the project.

### Precision of Control Calls

**Fig. 4:**
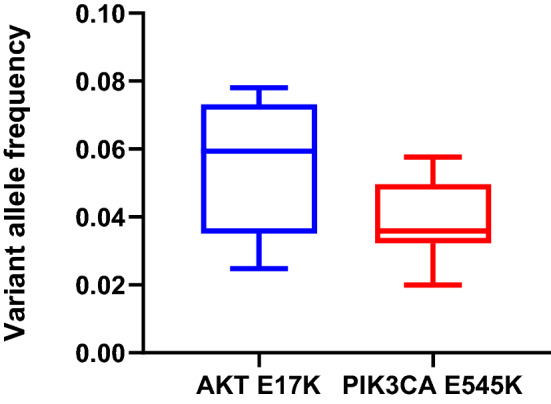
Box and whisker plot of repeated measures of VAF of known low VAF variants in a HD753 control. Known VAF is 0.054 for the AKT E17K mutation and 0.05 for the PIK3CA E545K mutation. *VAF* variant allele frequency

In order to understand the ability of the assay to detect low variant allele frequencies (VAFs), assessment of the VAF was performed for two known low VAF mutations in the HD753 cell line (Fig. 4). This cell line has validated mutations in *AKT1* (E17K, chr14: 105246551C > T), with a VAF of 0.05, and in *PIK3CA* (E545K, chr3: 178936091G > A), with a VAF of 0.056. The same control was run across 12 runs, with *AKT1* median VAF = 0.059 (IQR 0.037–0.072) and *PIK3CA* median VAF = 0.036 (IQR 0.033–0.0493).

### Copy-Number Calls

**Fig. 5:**
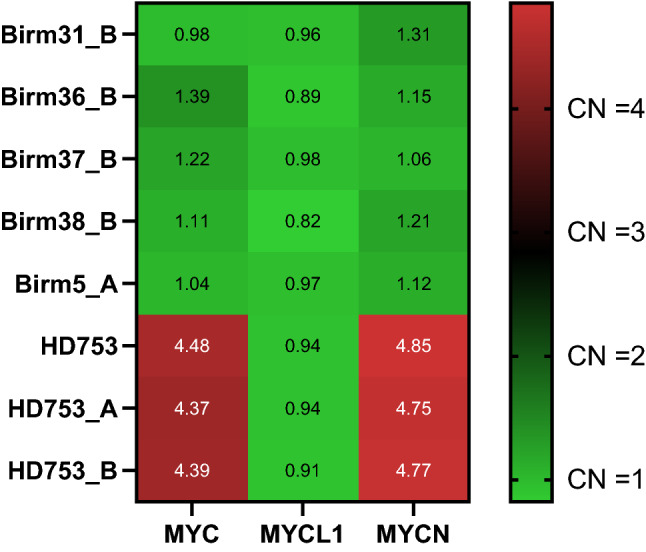
Copy-number (CN) heat map of detected CN in HD753 samples with known amplification in MYCN and MYC. Sample IDs on *y*-axis

A subset of 24 samples underwent copy-number calling with the Craft pipeline. A variety of copy-number gains and losses were detected in the 520 genes profiled on the TSO500 panel. The HD753 control was used to determine whether the observed copy-number calls (Fig. 5) correlated with known copy-number changes: amplifications in *MET* (CNV = 4.5) and *MYC-N* (CNV = 9.5). The amplification in *MET* was observed in all control samples, with an average copy number of 4, and in *MYC-N*, with an average copy number of 9.1. No whole-gene deletions were present in the control samples, but were observed in a variety of samples in the tumour cohort.

### Structural Variant Calls

The HD753 control is known to have a variety of structural variants, including an *SLC34A2*/*ROS* fusion (VAF = 5.6%) and *CCDC6*/*RET* fusion (VAF = 5.0%). With use of a custom pipeline, there was evidence for detection of both fusions: 7/506 reads supported the *SLC34A2/ROS* fusion and 5/498 reads supported the *CCDC6/RET* fusion. A variety of structural variants were observed in the tumour cohort. In addition, long indels were successfully detected by the Manta pipeline, specifically a 14-bp deletion in *EGFR* (NM_005228.5:c.2235_2249del), known to be present in the HD753 control.

### Tumour Mutational Burden and Microsatellite Instability

**Fig. 6:**
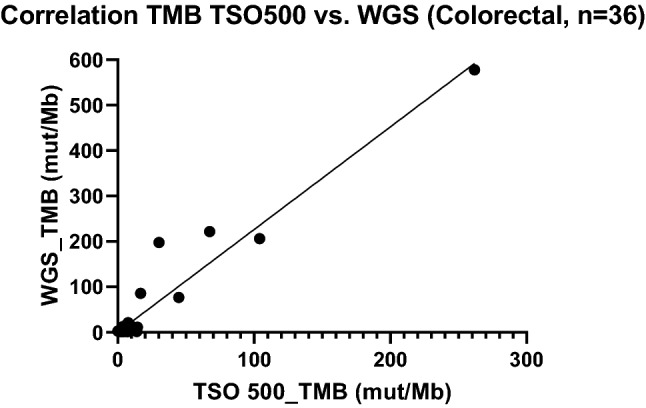
Correlation between TMB as measured by TSO500 assay vs. PCR-free WGS on colorectal samples (*R*^2^ = 0.98, *p* < 0.001). *PCR* polymerase chain reaction, *TMB* tumour mutational burden, *TSO500* TruSight Oncology 500, *WGS* whole-genome sequencing

**Fig. 7:**
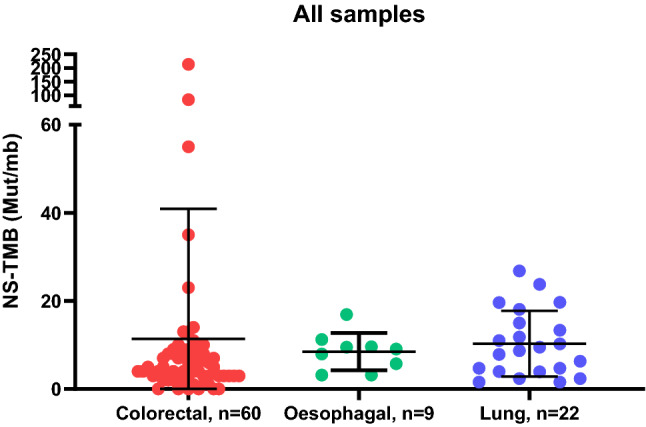
Distribution of mutations/megabase of DNA by tumour type. *Y*-axis: NS-TMB in mutations per megabase of DNA. *NS-TMB* non-synonymous tumour mutational burden

**Fig. 8:**
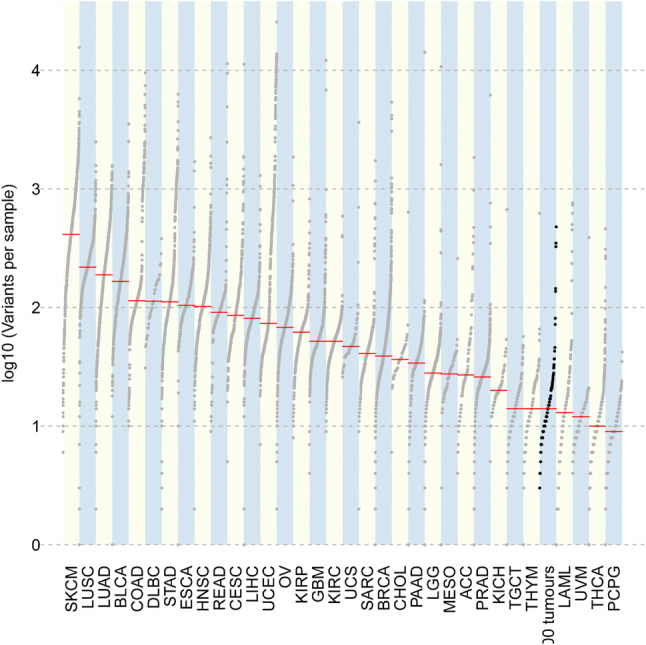
Plot of mutational burden in TSO500 trial tumour set as compared to tumours from TCGA. TSO500 trial tumour set shown in black. *Y-axis* Log10(variants per sample), *TCGA* The Cancer Genome Atlas, *TSO500* TruSight Oncology 500

TMB calling was successfully performed in 107/108 samples. The one failure was a sample with very poor quality that failed hybridisation. There was good correlation between TMB determined by TSO500 and WGS (*R*^2^ = 0.9, Fig. 6). The median TMB was 8.6 mut/Mb (range 0.85–325 mut/Mb, Fig. 7). Several known hypermutant tumour samples were deliberately run first, including a somatic *POLE* mutant colorectal cancer (reported TMB 261.71 mut/Mb in WGS sample), a somatic *MLH1* mutant colorectal cancer (reported TMB 67.43 mut/Mb) and a somatic *MSH6* mutant colorectal cancer (reported TMB 104.0 mut/Mb). As the HD753 control was run in each experiment, we compared the reproducibility of the TMB measurement for this control sample. There was a median TMB of 311 mut/Mb (range 289–325) in the HD753 control, a variance in TMB score of ± 5%. Comparison to TCGA tumour cohorts was performed in mafTools and is shown in Fig. 8.

**Fig. 9:**
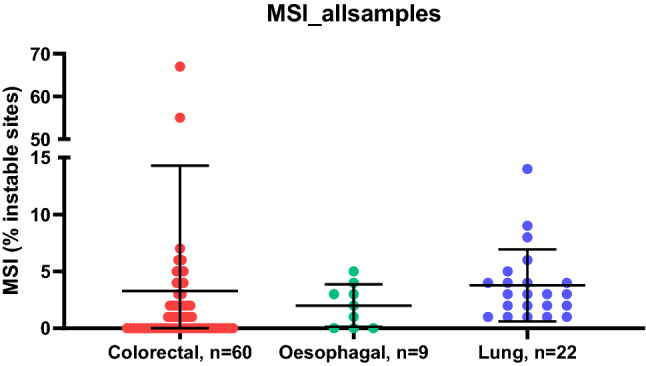
Percentage of microsatellite unstable probes against tumour type. Colorectal = red, oesophageal = green and lung = blue. *MSI* microsatellite instability

For MSI (Fig. 9), the threshold for classification as microsatellite instability high (MSI-H) was > 10% of microsatellite sites being unstable. Using this threshold, both known somatic mismatch repair mutant (MLH1 and MSH6) cancers were MSI-H, with 55% and 67% of sites being unstable, respectively. Reassuringly, the *POLE* mutant cancer had 2% of MSI sites being unstable, meaning it was microsatellite stable (MSS), as is typical in *POLE* mutant cancer.

### RNA Fusions

RNA fusion analysis was carried out on 13 samples, of which six had known fusions. Fusions were detected between *ETV6* and *NTRK3* (three samples), *RBPMS* and *NTRK3* (one sample), *EML4* and *ALK* (one sample) and *TG* and *RET* (one sample). All fusions that had previously been identified by fluorescence in situ hybridisation (FISH) were detected using this methodology. A fusion was detected in one sample between *ETV6* and *NTRK3* that had not been identified via FISH; however, the fusion was supported by 12,627 reads in the sequencing run, which we felt was unlikely to be a false positive, and therefore, we labelled it as a true fusion.

### Clinical Actionability

In order to recover as many clinically actionable variants as possible, mutational calls were fed into the OncoDNA OncoKDM (Gosselies, Belgium) and PierianDx CGW (St. Louis, MO, USA) pipelines. OncoDNA was provided with the vcf files coming from the Illumina pipeline, and PierianDx started the analysis directly from the FASTQ files. Also, in order to take an overview of pathway mutations and potential targets, the *OncogenicPathways* function of *mafTools* was used to generate a list of druggable pathways. The rat associated sarcoma pathway had 39/85 genes mutated (45.9%), the phosphoinositide 3-kinase pathway had 20/29 (69.0%), and transforming growth factor beta had 6/7 (85.7%).

Using this combined approach, more than 72 tier 1 variants and more than 297 tier 2 variants were identified between the two pipelines. Twenty-one samples were classified as TMB high, 19 as TMB medium and the remainder (64) TMB-low. Clinical trials could also be identified for all samples, with a median of 22 (range 4–105) trials suggested per sample. The most common actionable mutations observed were *BRAF* p.V600E (18 samples), *KRAS* p.G13D (14 samples) and *KRAS* p.G12D (12 samples). For other tiers, there were 8175 tier 3 mutations and 17,649 tier 4 mutations detected.

PierianDx and OncoKDM pipelines were not directly comparable in this study because of the differing inputs (FASTQ for PierianDx, vcf for OncoKDM), but the combination of both platforms provided a comprehensive variant overview.

## Discussion

We utilised the TSO500 assay in order to understand its utility and accuracy in determining both the TMB and druggable mutation calls in cancer. One of the key challenges with patient testing [23] is the ability to take a patient biopsy sample with limited input material and produce sequencing data and mutational calls of sufficient quality in order to make decisions on target selection and drug therapy [24].

The assay was designed in its first iteration to measure TMB as a surrogate marker for response to anti–PD-1 immunotherapy, as multiple studies have shown a correlation between TMB and response to this type of therapy [8, 13]. The TSO500 assay performs well in this respect, with accurate measurement of TMB when compared to WGS. Taking a threshold of 10 mut/Mb as “TMB high” (i.e. that which would have benefit for immunotherapy), we found that the TSO500 assay was able to classify samples with 100% accuracy. The precision of the calls varies at the extremes of TMB values, undoubtedly as a factor of panel size in calling TMB at extremely high levels. We conclude that the TSO500 pipeline is usable in the clinical determination of TMB status across a range of clinical sample types and DNA inputs.

We successfully detected MSI in all samples that were known to be MSI-H using TSO500. MSI detection using NGS has been shown to be feasible [25] previously, using a variety of software solutions, usually relying on off-target reads [26], but other assays have used dedicated MSI probes (like the TSO500). We have found that the performance of this approach is variable, as the probes are vulnerable to drop out in FFPE samples. We propose that TMB instead may be a good surrogate biomarker for MSI, as a range of 30–80 mut/Mb is typically seen in MSI tumours, as opposed to MSS *POLE*/*POLD1* tumours, which typically have greater than 150 mut/Mb.

A key requirement for clinical specimens is the ability to process low-input specimens as well as the ability to detect the low VAFs associated with these specimens [27]. Reassuringly, we found that the TSO500 assay performed well at its recommended input concentration and also below these levels. Within our control samples with known VAF (of approximately VAF = 0.05), we determined that there was good precision and reproducibility with minimal variability. Another advantage to tolerance of low sample input is the possibility of using input levels seen in circulating tumour DNA (ctDNA), which are typically 1 ng/mL plasma in most cancers. This would allow derivation of blood TMB [28], which has been shown to be a better biomarker of response in PD-1/PD-L1 inhibitor therapy. The assay is also performed at sufficiently high read depth to allow calculation of clonal TMB [29, 30], another marker associated with more accurate identification of potential response to immunotherapy.

In terms of identifying druggable mutations for targeted therapy selection, the TSO pipeline presents an attractive platform, especially when coupled with a clinical annotation engine such as the two used here (OncoKDM and PierianDx CGW [31]). We found good correlation between mutations detected in WGS experiments, and the identification of druggable mutations was made straightforward by the use of integrated clinical pipelines to produce reproducible data.

CNVs, especially amplifications, represent important therapeutic targets. The TSO500 assay detected the known amplifications in a control sample, meaning that patients can potentially undergo therapeutic targeting. A unique advantage of the TSO500 system is the ability of a partner targeted RNA-seq assay that can detect RNA fusions. We found that the assay reliably detected *NTRK* [32], *ALK* [33] and *RET* [34] fusions that had previously been identified by FISH, as well as a novel fusion not previously detected using other technologies. Intriguingly, we also successfully detected known fusions at the DNA level de novo in the HD753 control sample, suggesting that this methodology may also be valid for future use, although DNA-based fusion calling has a high false negative rate. Fusion genes represent good drug targets, and a number of novel agents [19, 32] have been shown to be active against fusion genes. Detection of circulating RNA for these fusion genes may also be possible [35] using this assay and could be explored further.

Direct comparison of analysis pipelines can be difficult, particularly when the data files fed into the systems have different sources. Although PierianDx hosts Illumina’s secondary analysis for the TSO500 panel and OncoDNA were provided with UMI collapsed vcf files from an Illumina pipeline, there is no indication that the pipeline versions were the same. This, along with the possibility that the same selection criteria and filter settings were not used by the two systems, could account for the fact that the PierianDx CGW platform returned more clinical associations and clinical trials, but this was not investigated here. For the purpose of this study, this difference was not explored as it was clear that both systems were suitable for determining clinical actionability in a routine clinical setting.

The UK 100,000 Genome project has recently been completed, and analysis and reporting are ongoing. The use of WGS for tumour-normal pairs using fresh frozen material still has significant challenges from a cost perspective as well as the practicalities of obtaining fresh frozen tissue over readily available paraffin-embedded material. The TSO500 assay costs approximately one third of the price of a WGS assay, requires no germline DNA control, allows RNA fusion detection and can be implemented on bench-top sequencers. Its main limitations include more laborious library preparation and enrichment chemistry that is vulnerable to drop out.

In conclusion, we believe that the TSO500 assay offers a cost-effective, accurate, pan-cancer assay that can derive SNP, CNV, and gene fusion information across the majority of cancers using a standardised pipeline and therefore is suitable for routine use in precision oncology as a comprehensive genomic profiling solution. In addition, several commercial decision support tools, two of which were successfully tested here, are available to assist in the interpretation of the increased number of variants that are derived from such a large gene set.

## Electronic supplementary material

Below is the link to the electronic supplementary material.

Supplementary file1 (PDF 49 kb)

## Data Availability

All data are available in the European Genome Phenome Archive.
