# Correction to: Use of an Integrated Pan-Cancer Oncology Enrichment Next-Generation Sequencing Assay to Measure Tumour Mutational Burden and Detect Clinically Actionable Variants

**DOI:** 10.1007/s40291-020-00479-2

**Published:** 2020-06-22

**Authors:** Valerie Pestinger, Matthew Smith, Toju Sillo, John M. Findlay, Jean‑Francois Laes, Gerald Martin, Gary Middleton, Phillipe Taniere, Andrew D. Beggs

**Affiliations:** 1 Surgical Research Laboratory, Institute of Cancer and Genomic Sciences, University of Birmingham, Vincent Drive, Birmingham, B15 2TT UK; 2 Queen Elizabeth Hospital Birmingham, Birmingham, UK; 3 Northern Devon Healthcare NHS Trust, Barnstaple, UK; 4 OncoDNA, Gosselies, Belgium; 5 PierianDx, St. Louis, MO USA; 6 Institute of Immunology and Immunotherapy, University of Birmingham, Birmingham, UK

## Correction to: Molecular Diagnosis & Therapy (2020) 24:339–349 10.1007/s40291-020-00462-x

This article was originally published under a [CC BY NC 4.0] license, but has now been made available under a Creative Commons Attribution 4.0 International License (https://creativecommons.org/licenses/by/4.0/), which permits use, sharing, adaptation, distribution and reproduction in any medium or format, as long as you give appropriate credit to the original author(s) and the source, provide a link to the Creative Commons licence, and indicate if changes were made. The PDF and HTML versions of the paper have been modified accordingly.

