# Supplementary material for: Use of an Integrated Pan-Cancer Oncology Enrichment Next-Generation Sequencing Assay to Measure Tumour Mutational Burden and Detect Clinically Actionable Variants
Source: Mol Diagn Ther. 2020 Apr 18;24(3):339–49. doi: 10.1007/s40291-020-00462-x (PMC7264086; doi:10.1007/s40291-020-00462-x)
Supplement: Supplementary file 1 — Supplementary file1 (PDF 49 kb) [file 40291_2020_462_MOESM1_ESM.pdf]

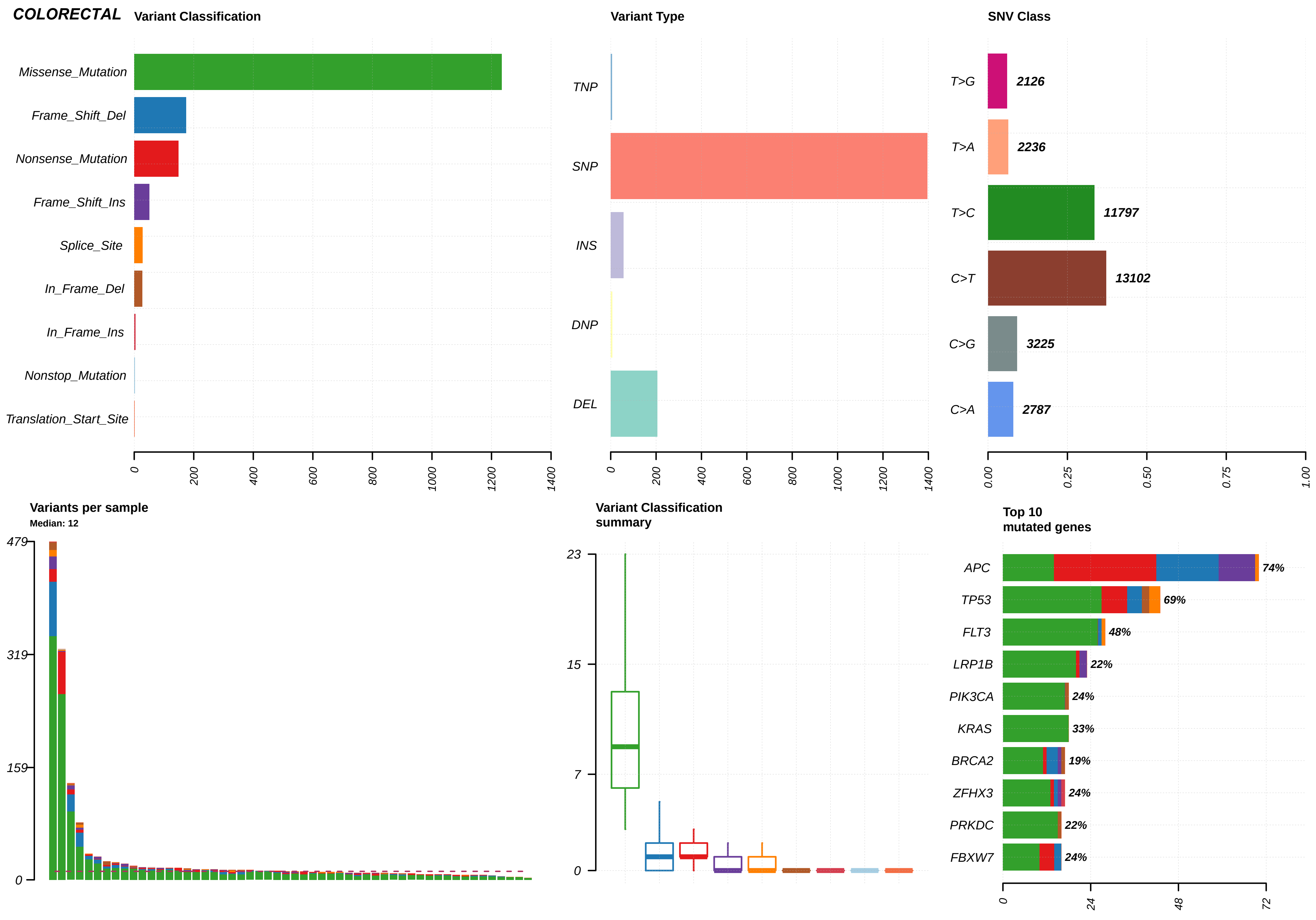
COLORECTAL
Variant Classification
Missense_Mutation
Frame_Shift_Del
Nonsense_Mutation
Frame_Shift_Ins
Splice_Site
In_Frame_Del
In_Frame_Ins
Nonstop_Mutation
Translation_Start_Site
0 200 400 600 800 1000 1200 1400
Variant Type
TNP
SNP
INS
DNP
DEL
0 200 400 600 800 1000 1200 1400
SNV Class
T>G 2126
T>A 2236
T>C 11797
C>T 13102
C>G 3225
C>A 2787
0.00 0.25 0.50 0.75 1.00
Variants per sample
Median: 12
479
319
159
0
Variant Classification summary
23
15
7
0
Top 10 mutated genes
APC 74%
TP53 69%
FLT3 48%
LRP1B 22%
PIK3CA 24%
KRAS 33%
BRCA2 19%
ZFHX3 24%
PRKDC 22%
FBXW7 24%
0 24 48 72

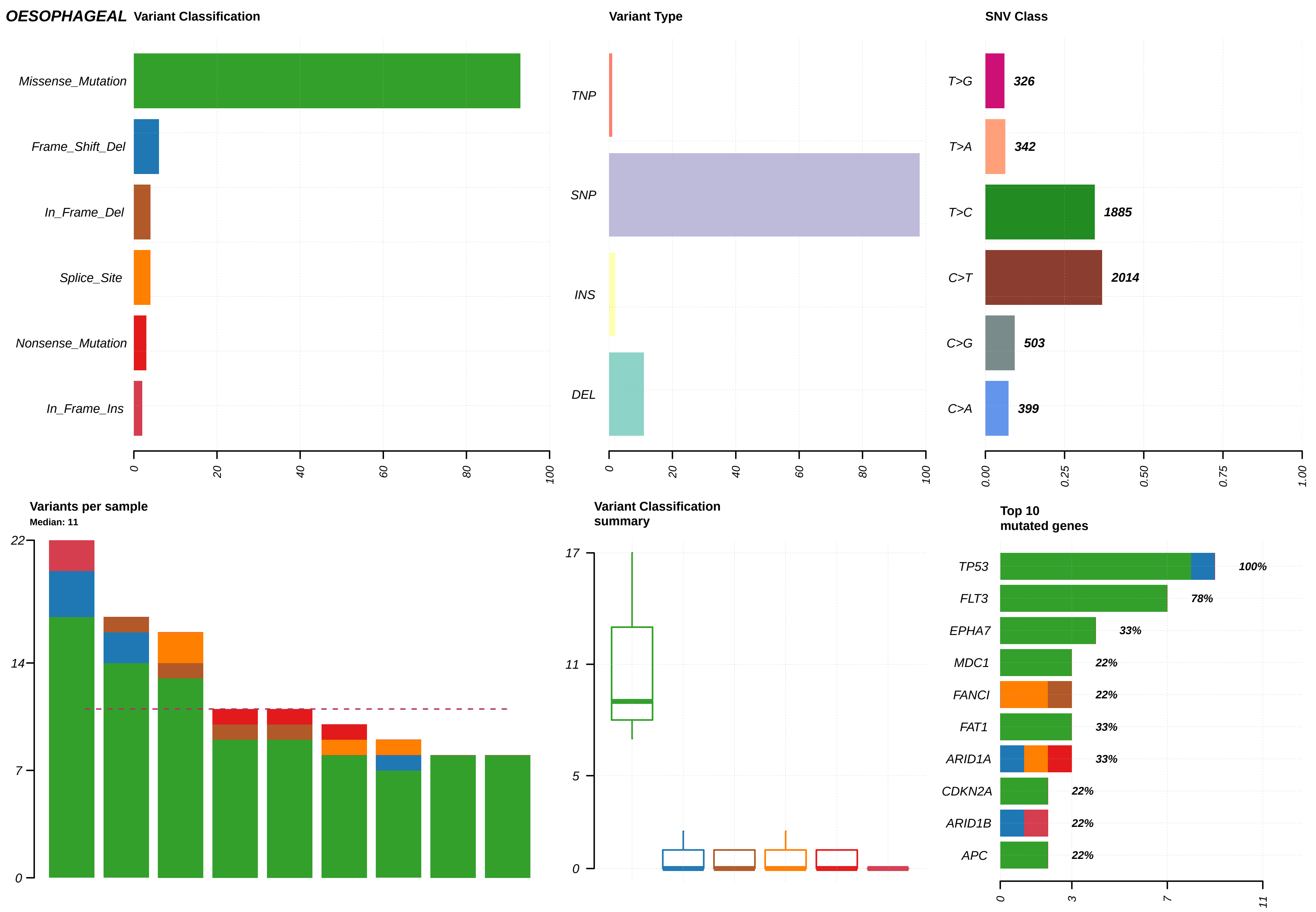
OESOPHAGEAL
Variant Classification
Missense_Mutation
Frame_Shift_Del
In_Frame_Del
Splice_Site
Nonsense_Mutation
In_Frame_Ins
0
20
40
60
80
100
Variant Type
TNP
SNP
INS
DEL
0
20
40
60
80
100
SNV Class
T>G
326
T>A
342
T>C
1885
C>T
2014
C>G
503
C>A
399
0.00
0.25
0.50
0.75
1.00
Variants per sample
Median: 11
22
14
7
0
Variant Classification
summary
17
11
5
0
Top 10
mutated genes
TP53
100%
FLT3
78%
EPHA7
33%
MDC1
22%
FANCI
22%
FAT1
33%
ARID1A
33%
CDKN2A
22%
ARID1B
22%
APC
22%
0
3
7
11

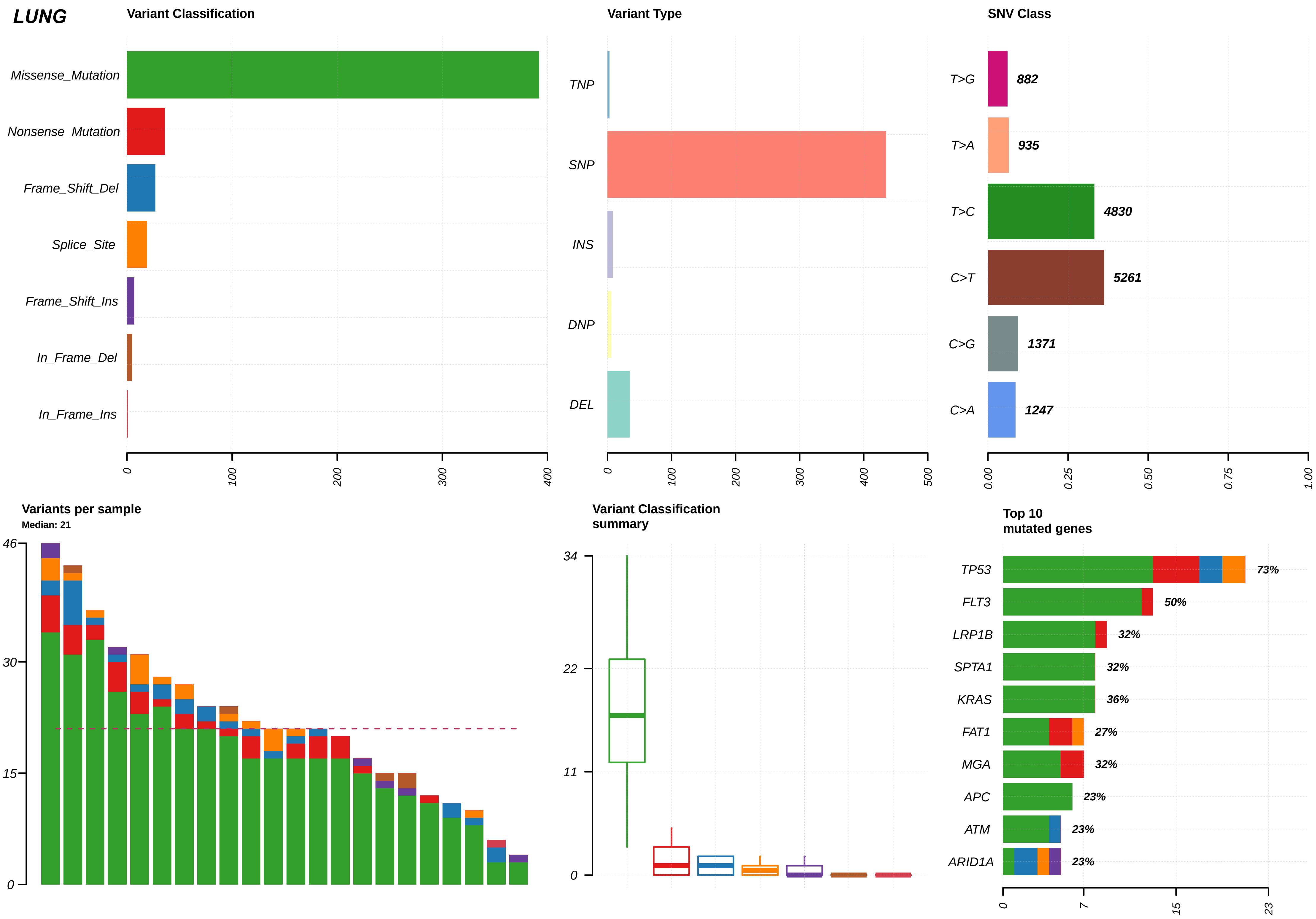
LUNG
Variant Classification
Missense_Mutation
Nonsense_Mutation
Frame_Shift_Del
Splice_Site
Frame_Shift_Ins
In_Frame_Del
In_Frame_Ins
0
100
200
300
400
Variant Type
TNP
SNP
INS
DNP
DEL
0
100
200
300
400
500
SNV Class
T>G 882
T>A 935
T>C 4830
C>T 5261
C>G 1371
C>A 1247
0.00
0.25
0.50
0.75
1.00
Variants per sample
Median: 21
46
30
15
0
Variant Classification
summary
34
22
11
0
Top 10
mutated genes
TP53 73%
FLT3 50%
LRP1B 32%
SPTA1 32%
KRAS 36%
FAT1 27%
MGA 32%
APC 23%
ATM 23%
ARID1A 23%
0
7
15
23
